# Development and validation of the Spatial Navigation and Memory Questionnaire (SPAM)

**DOI:** 10.1002/dad2.70441

**Published:** 2026-08-11

**Authors:** Monique S. Boord, Sol Morrissey, Hannah A. D. Keage, Victor R. Schinazi, Michael Hornberger, Stephanie Wong

**Affiliations:** ^1^ College of Human Sciences and Culture Flinders University Adelaide South Australia Australia; ^2^ Alliance for Research in Exercise, Nutrition and Activity Adelaide University Adelaide South Australia Australia; ^3^ Schulich School of Engineering, University of Calgary, Calgary Alberta Canada; ^4^ School of Psychology Adelaide University Adelaide South Australia Australia; ^5^ Future Health Technologies Singapore‐ETH Centre Campus for Research Excellence and Technological Enterprise (CREATE) Singapore Singapore; ^6^ Department of Psychology Bond University Gold Coast Queensland Australia; ^7^ Clinical Neurosciences Faculty of Medicine University of Southampton Southampton UK

**Keywords:** aging, memory, questionnaire, spatial navigation

## Abstract

**INTRODUCTION:**

Spatial navigation deficits may provide a sensitive and specific marker of early Alzheimer's disease pathology. However, few spatial navigation assessment tools are available for clinical practice that are accessible, time efficient, and sensitive to cognitive aging.

**METHODS:**

Exploratory (*n = *470) and confirmatory factor analyses (*n =* 470) were conducted to establish the factor structure of the Spatial Navigation and Memory Questionnaire (SPAM) in healthy older adults. A subset of participants (*n =* 445) completed the Virtual Supermarket Test (VST) to assess convergent validity. Normative data were reported stratified by age and sex.

**RESULTS:**

A two‐factor structure reflecting spatial navigation and memory for both subscales of the SPAM was supported, with acceptable to good internal consistency across factors. SPAM spatial navigation scores and VST performance were significantly correlated.

**DISCUSSION:**

The SPAM demonstrates a clear and interpretable factor structure, acceptable model fit, and preliminary evidence of validity with an objective measure of spatial navigation performance.

## Highlights

- The Spatial Navigation and Memory Questionnaire (SPAM) adequately captures domains of spatial navigation and memory function.
- Poor recent self‐reported navigation is associated with poor objective performance.
- The SPAM structure allows interpretation of lifetime and recent subscale scores.
- The SPAM is a practical and time‐efficient evaluation of spatial navigation and memory.

## 1 INTRODUCTION

Accumulating evidence suggests that tests of spatial navigation are more sensitive and specific to early Alzheimer's disease (AD) pathology^1^, ^2^, ^3^, ^4^ and that spatial navigation deficits present years preceding diagnosis.^3^ Spatial navigation refers to the ability to plan and maintain a trajectory from one location to another.^5^ Successful navigation of our environment relies on two co‐dependent spatial reference frames: allocentric and egocentric.^5^, ^6^, ^7^ Egocentric navigation involves wayfinding from the viewpoint of the navigator (self to object) and is generally used when following a familiar route.^5^, ^6^, ^7^, ^8^ In comparison, allocentric navigation strategies tend to be used in novel environments and rely on the navigator's perception of spatial relationships between landmarks (object to object^6^, ^7^, ^8^).

Neuroimaging evidence indicates that spatial navigation deficits are associated with structural integrity of medial temporal lobe regions, which show early neuronal loss in AD.^9^ Difficulty navigating unfamiliar environments and subtle disorientation are common in early AD and mild cognitive impairment, often preceding noticeable memory loss.^1^ Progression of AD is associated with worsening navigational deficits; for example, being unable to navigate familiar locations.^6^ A growing body of evidence^1^, ^2^, ^3^, ^4^ therefore suggests that assessing spatial navigation may offer a more sensitive approach in detecting subtle and early changes associated with AD. Beyond its clinical relevance for AD, however, some aspects of spatial navigation decline with healthy aging.^6^ A large study with individuals aged 8 to 100 years showed spatial navigation performance to linearly decrease with age, with 70‐ to 100‐year‐olds showing the poorest performance on a path route task.^10^ Performance also decreased with age on a landmark and allocentric task, and besides the younger group (8–40 years) outperforming the older age groups (40–100 years), no further age‐related decline within the latter group was reported in an egocentric task.^10^ Egocentric navigation appears to remain relatively stable, while allocentric navigation decreases with increasing age.^11^

Current methods of spatial navigation testing include questionnaires^12^ and real world^13^ or virtual reality^14^ assessments. However, few tools are available for clinical practice that are both accessible and time efficient. Existing measures were not originally designed for clinical application and lack clinically relevant features for early AD detection, including comparison of premorbid and recent ability (allowing within‐subject comparison), assessment of both egocentric and allocentric navigation, safeguarding issues, and direct assessment of memory, particularly attentional memory (memory processes that are dependent on effective allocation of attentional resources) which is also frequently reported in early cognitive decline. Specific questionnaires tailored for prodromal or preclinical AD are needed and should incorporate both memory and spatial navigation items, as the two are the earliest domains to be affected in AD. While normal age‐related changes in memory are well characterized,^15^, ^16^, ^17^ fewer studies have established normal versus pathological changes in spatial navigation.

**RESEARCH IN CONTEXT**

1. **Systematic review**: Previous evidence suggests that spatial navigation tests are more sensitive and specific to early Alzheimer's disease pathology. Few spatial navigation tools are available for clinical practice that are accessible, time efficient, and include clinically relevant items or normative benchmarks.
2. **Interpretation**: Our findings position the Spatial Navigation and Memory Questionnaire (SPAM) as an appropriate assessment of spatial navigation and memory abilities in heathy older adults. Findings also provide support for the convergent and divergent validity of the spatial component of the SPAM, and establish normative data to interpret scores in a range of assessment contexts for different sex and age groups in healthy older adults.
3. **Future directions**: Further research should validate the SPAM in clinical samples including those with mild cognitive impairment and dementia to further clarify its diagnostic application.

The Spatial Navigation and Memory Questionnaire (SPAM), a 30‐item self‐report and informant questionnaire assessing recent and lifetime (premorbid) spatial navigation and memory ability, was developed to address these needs. Although robust evidence is emerging for the role of spatial navigation deficits in preclinical AD, validation of tests for clinical use is required first in a cognitively healthy sample. Further, normative data and cut‐off scores are necessary to allow for interpretation of results at an individual patient level. The aims of the current study were to:

1. Explore the factor structure of the SPAM.
2. Explore the convergent validity of the SPAM using performance on an objective test of spatial navigation, the Virtual Supermarket Test (VST).^2^, ^18^
3. Establish normative data for the SPAM stratified by age and sex in a healthy sample.

## 2 METHOD

### 2.1 Sample

Nine hundred forty participants from the UK‐based Driver Effect of Cognitive Impairment and Spatial Orientation and Navigation (DECISION) study who completed the SPAM were included (for further details regarding the DECISION cohort refer to Morrissey et al.^19^, ^20^, ^21^). A subset (*n =* 445) completed the VST. Age and sex were self‐reported. The sample was divided into two age‐ and sex‐matched groups: one (*n =* 470) used for exploratory factor analysis and the other (*n =* 470) for confirmatory factor analysis.

### 2.2 Procedure

Participants were recruited via online or media advertisement and were included if they were ≥ 65 years, had a current driving license, and drove at least once per week. Participants were excluded if they did not drive regularly, had a medical condition that contraindicated driving (including untreated significant visual or physical impairments), a diagnosis of major neuropsychiatric disorder (learning disabilities, substance abuse, major depression, schizophrenia), mild cognitive impairment or dementia, took medications for dementia, or had high alcohol consumption (> 45 units per week).

Participants completed the measures through an online survey (https://mantal.co.uk) and cognitive testing platform (NeurOn; https://neuropsychology.online/) using their own devices (tablet, laptop, or desktop computer).

### 2.3 Measures

#### 2.3.1 SPAM

The SPAM was created in 2019 using a two‐round Delphi approach. The purpose of the SPAM was to detect subtle changes in memory, attention, and spatial navigation, tailored for identifying signs of preclinical AD and to assess functional changes. Requirements included: ability for self‐administration; short administration (< 10 minutes); assessment of memory and spatial navigation symptoms with items targeting everyday changes in memory, attention, and spatial navigation, as well as potential safeguarding issues (not symptoms per se but how symptoms might affect patient safety issues); items assessing premorbid abilities versus recent changes to allow for comparison pre and post disease onset.

The SPAM underwent five stages of development by a panel (consisting of a neurologist, a cognitive neuroscientist, a clinical psychologist, two early career researchers, and a caregiver of a person with dementia): (1) review of existing scales, (2) identifying current shortcomings of existing scales, (3) item development individually, (4) item revision together, and (5) consensus on final items. Most items were informed by the Cambridge Behavioural Inventory‐Revised,^22^ Neuropsychiatric Inventory Questionnaire,^23^ the Cognitive Change Inventory,^24^ the Santa Barbara Sense of Direction Scale,^12^ and the Questionnaire on Everyday Navigational Ability.^25^ Finalized items underwent two rounds of feedback by the same panelists.

The SPAM contains 30 self‐report items assessing recent (over the previous 3 months) and lifetime spatial navigation and memory abilities (see Table S1 in supporting information). Spatial navigation items evaluate general navigation behavior, egocentric and allocentric navigation strategy use, spatial memory, and disorientation. Memory items assess recall of events, names, and faces; learning new information; and attentional memory problems. The lifetime subscale comprises 12 items (6 memory, e.g., “I always had a good memory for events”; 6 spatial navigation, e.g., “I have always been good at giving directions”), which were rated on a scale of 0 to 4 (strongly disagree, disagree, neither agree nor disagree, agree, strongly agree). The recent subscale comprises 18 items (9 memory, e.g., “I have difficulty learning new information”; 9 spatial navigation, e.g., “I have been unsure how to get to familiar places in my neighborhood”), which were rated on a scale of 0 to 4 (daily, a few times a week, a few times a month, rarely, never). All items on both subscales of the SPAM were reverse scored, such that higher scores indicated poorer ability.

#### 2.3.2 VST

Participants were presented with 14 first‐person perspective video trials of a shopping cart moving through a virtual supermarket and after each trial were prompted to indicate the starting direction (egocentric navigation) and reproduce the travel route on a map of the supermarket (allocentric navigation).^2^, ^18^ The VST is shown to discriminate between AD and non‐AD dementias,^2^ classify genetic risk for AD,^26^ and shows good test–retest reliability for egocentric navigation.^27^ Egocentric and allocentric errors were calculated for analysis. Detailed VST procedures are described in Supplementary Text A and Figure S1 in supporting information.

### 2.4 Analytical approach

#### 2.4.1 Exploratory factor analysis

Exploratory factor analysis of the SPAM was conducted in JAMOVI (version 2.2.5). The Kaiser–Meyer–Olkin (KMO) measure of sampling adequacy and Bartlett test of sphericity were used to check assumptions and data suitability. Sphericity was satisfied if the Bartlett test was significant (*P <* .05). KMO values between 0.5 and 0.7 were considered adequate, 0.7 and 0.9 good, and 0.9 and 1.0 excellent for sampling adequacy.^28^, ^29^ Retained factors were determined through Kaiser criterion (eigenvalues > 1) and scree plot inspection. Factor extraction was completed using the maximum likelihood method. An oblique rotation method was applied (promax), and factor loadings were investigated to identify which items load significantly on to each factor.

#### 2.4.2 Confirmatory factor analysis

Latent factors identified in the exploratory factor analysis of the SPAM were examined through confirmatory factor analysis. Factor structure was derived through consensus agreement. Model fit was assessed using the root mean square error of approximation (RMSEA) and comparative fit index (CFI), with RMSEA < .05 and CFI > .90 indicating good model fit.^30^ Model fit indices, factor loadings for each item on its respective factor, and error variances were extracted for reporting.

#### 2.4.3 Reliability/validity

Internal consistency was assessed using Cronbach alpha. Pearson correlations were performed to assess convergent validity between the SPAM and performance on the VST. Correlation analyses were conducted separately for recent spatial navigation, recent memory, lifetime spatial navigation, lifetime memory subscales, and allocentric and egocentric navigation error scores on the VST.

#### 2.4.4 Normative data

Participants were stratified by age (60–69, 70–79, 80–89) and sex (M, F). Means and standard deviations were reported for total scores on the SPAM subscales (lifetime and recent) and further split by spatial and memory questions within each subscale. A series of two‐way analyses of variance (ANOVA), with sex and age group as between‐subject factors assessed differences in SPAM scores split by spatial and memory questions within each subscale (see Supplementary Text B in supporting information). Difference scores between recent and lifetime scales are reported in Supplementary Text C in supporting information and Table 2.

## 3 RESULTS

### 3.1 Sample characteristics

Sample demographics are listed in Table S3 in supporting information. The mean age of participants in the EFA sample was 71.3 years and 46% were male. The mean age of participants in the CFA sample was 70.9 years and 42% were male. Participants in both samples were generally well educated with approximately one third of both samples having obtained a bachelor's degree, approximately one quarter obtained postgraduate qualifications, and < 1% having obtained less than secondary school education. There were no significant differences in age (*t =* –1.45, *P *= .147), sex (χ^2^ *= *1.24, *P *= .265), or highest level of education (χ^2^ = 9.39, *P *= .226) between samples.

### 3.2 Exploratory factor analysis

#### 3.2.1 Lifetime

The Kaiser criterion and scree plot examination suggested two factors for extraction. Both factors captured 45.5% of the variance and were positively correlated with each other (*r *= .51). Factor loading examination suggested one factor reflects spatial navigation ability while the second factor reflects memory. Internal consistency for the spatial navigation (α* =* .866) and memory (α* =* .757) factors was good. One item (Q10 “I usually followed landmarks when finding my way around”) was removed due to not loading onto either factor. Factor loadings and communalities are provided in Table 1.

**TABLE 1:** Factor loadings for spatial navigation and memory ability over the lifespan on the SPAM.

SPAM item	Factor 1 spatial navigation	Factor 2 memory	Communality
Q7. I have always been good at finding my way around, even without maps or GPS	0.912		0.248
Q12. I rarely got disoriented as to my location	0.884		0.225
Q9. I would rarely get lost in new places, for example on holiday or in an unknown town	0.822		0.354
Q11. I had an internal map (in my head) of my environment when finding my way around	0.815		0.379
Q8. I have always been good at giving directions	0.585		0.588
Q2. I always had a good memory for names		0.739	0.560
Q1. I always had a good memory for events		0.670	0.518
Q4. I rarely needed to keep lists to remember things		0.637	0.596
Q5. I rarely got distracted when trying to remember something		0.591	0.671
Q3. I always had a good memory for faces		0.497	0.733
Q6. My mind rarely drifted during conversations or meetings		0.449	0.778

*Note*: *N* = 470.

Abbreviations: GPS, global positioning system; SPAM, Spatial Navigation and Memory Questionnaire.

#### 3.2.2 Recent

The Kaiser criterion and scree plot examination suggested two factors for extraction. Both factors jointly captured 30.9% of the variance and were positively correlated with each other (*r *= .36). Factor loading examination suggests one factor reflects memory while the second reflects spatial navigation ability. Internal consistency for the spatial navigation (α* =* .69) and memory (α = .79) factors was good. Three items (Q7 “I have been unsure where the rooms are in my home”; Q8 “I have been unsure how to get to familiar places in my neighborhood”; Q18 “I have relied on my GPS to navigate”) were removed due to not loading onto either factor. Factor loadings and communalities are provided in Table 2.

**TABLE 2:** Factor loadings for spatial navigation and memory ability over the last 3 months (recent) on the SPAM.

SPAM item	Factor 1 memory	Factor 2 spatial navigation	Communality
Q2. I have difficulty remembering recent events	0.756		0.436
Q4. I struggle to remember appointments	0.675		0.532
Q5. I forget what I was looking for when going to another room	0.587		0.65
Q1. I have difficulty learning new information	0.571		0.595
Q6. I struggle to remember where things belong in the house	0.548		0.695
Q3. I have difficulty remembering events from long ago	0.528		0.719
Q13. I have forgotten to switch off an appliance (such as the oven)	0.416		0.837
Q15. I have forgotten to take my medication, without being reminded	0.342		0.886
Q14. I have forgotten to eat or drink, without being reminded	0.320		0.910
Q9. I avoid going to new places as I am not sure how to find my way there		0.727	0.886
Q10. I avoid going to places in the dark, as I am getting lost more easily		0.702	0.91
Q12. If there is a roadblock or deviation in my journey, I have problems finding another route without help		0.576	0.533
Q11. I am looking more for landmarks, like church towers or distinctive buildings, to find my way around		0.477	0.544
Q17. I have been disoriented as to my location when driving		0.344	0.622
Q16. I have thought that I might not find my way back to my home when out		0.303	0.744

*Note*: *N* = 470.

Abbreviation: SPAM, Spatial Navigation and Memory Questionnaire.

### 3.3 Confirmatory factor analysis

#### 3.3.1 Lifetime

After removal of one spatial navigation item as per the lifetime subscale EFA above, and removal of a further item (Q8 “I have always been good at giving directions”) suggested by modification indices here, confirmatory factor analysis was conducted on 10 items (4 spatial navigation, 6 memory) in the lifetime subscale. The chi‐squared value for the model was significant: χ^2^(121) = 34, *P *< .001. Despite this, the RMSEA was  .074 with a 90% confidence interval (CI) of  .059 to  .088, indicating acceptable model fit. The CFI was  .948, indicating good model fit. Factor loadings are shown in Table 3. Factor loadings for the memory factor ranged from 0.413 to 0.671, all of which were statistically significant (*P *< .001). Significant loadings were demonstrated for the spatial navigation factor, ranging from 0.785 to 0.850. Factor covariance indicated a significant moderate positive relationship between the memory and spatial navigation factor (.327, *P *< .001).

**TABLE 3:** Confirmatory factor analysis factor loadings for lifetime spatial navigation and memory items.

Factor	Indicator	Estimate	SE	*Z*	*p*	Standard estimate
Spatial navigation	Q7_life	0.883	0.0438	20.18	< 0.001	0.805
	Q12_life	0.725	0.0360	20.17	< 0.001	0.805
	Q9_life	0.852	0.0389	21.89	< 0.001	0.850
	Q11_life	0.806	0.0414	19.46	< 0.001	0.785
Memory	Q2_life	0.746	0.0532	14.02	< 0.001	0.671
	Q1_life	0.683	0.0481	14.20	< 0.001	0.669
	Q4_life	0.705	0.0535	13.17	< 0.001	0.626
	Q5_life	0.623	0.0444	14.02	< 0.001	0.677
	Q3_life	0.481	0.0514	9.35	< 0.001	0.467
	Q6_life	0.430	0.0527	8.15	< 0.001	0.413

Abbreviation: SE, standard error.

#### 3.3.2 Recent

After removal of 3 spatial navigation items as per the recent subscale EFA above, confirmatory factor analysis was conducted on 15 items (6 spatial navigation, 9 memory) in the recent subscale. The chi‐squared value for the model was significant: χ^2^(275) = 89, *P *= < .001. Despite this, the RMSEA was  .067 with a 90% CI of  .058 to  .076, indicating reasonable model fit. The CFI was  .908, indicating good model fit. Factor loadings for memory and spatial items are shown in Table 4. Factor loadings for the memory factor ranged from 0.242 to 0.749, all of which were statistically significant (*P *< .001). Significant loadings were demonstrated, ranging from 0.195 to 0.739. Factor covariance indicated a significant moderate positive relationship between the memory and spatial navigation factor (.529, *P *< .001).

**TABLE 4:** Confirmatory factor analysis factor loadings for recent spatial navigation and memory items.

Factor	Indicator	Estimate	SE	*Z*	*p*	Standard estimate
Spatial navigation	Q9_recent	0.266	0.0231	11.51	< 0.001	0.585
	Q10_recent	0.405	0.0295	13.70	< 0.001	0.687
	Q12_recent	0.326	0.0286	11.40	< 0.001	0.578
	Q11_recent	0.395	0.0349	11.31	< 0.001	0.565
	Q16_recent	0.045	0.0071	6.34	<0.001	0.330
	Q17_recent	0.208	0.0266	7.81	< 0.001	0.413
Memory	Q2_recent	0.590	0.0348	16.89	< 0.001	0.739
	Q4_recent	0.427	0.0343	12.46	< 0.001	0.579
	Q5_recent	0.567	0.0441	12.85	< 0.001	0.593
	Q1_recent	0.476	0.0317	14.99	< 0.001	0.673
	Q6_recent	0.309	0.0292	10.57	< 0.001	0.503
	Q3_recent	0.364	0.0369	9.83	< 0.001	0.472
	Q13_recent	0.303	0.0279	10.85	<0 .001	0.518
	Q15_recent	0.249	0.0270	9.22	< 0.001	0.448
	Q14_recent	0.065	0.0169	3.82	<0.001	0.195

Abbreviation: SE, standard error.

### 3.4 Convergent validity

Weak positive correlations were found between recent spatial navigation and egocentric error (*r *= 0.157, *P *< .001) and allocentric error (*r *= 0.200, *P *< .001), such that greater difficulties with recent spatial navigation on the SPAM were associated with greater egocentric and allocentric navigation errors on the VST. Associations remained significant after Holm–Bonferroni correction. A weak negative correlation was found between recent memory and allocentric error (*r* = –0.115, *p* = .016) but did not survive corrections for multiple comparisons. No statistically significant correlations were identified between lifetime spatial navigation or lifetime memory subscales and either allocentric or egocentric errors on the VST.

### 3.5 Normative data

Normative data for both subscales of the SPAM and the two factors within each subscale (lifetime; 10 items and recent; 15 items) are presented in Table 5, with scores stratified by age and sex.

**TABLE 5:** Average SPAM scores stratified by age and sex for the SPAM subscales.

	60–69 years		70–79 years		80–89 years	
	M (*n =* 174)	F (*n =* 262)	M (*n =* 199)	F (*n =* 238)	M (*n =* 46)	F (*n =* 21)
**Lifetime memory** (*M*, SD) Max score = 24	11.2 (2.42)	11.0 (2.49)	11.3 (2.49)	11.2 (2.35)	11.9 (2.77)	11.7 (2.46)
**Lifetime spatial** (*M*, SD) Max score = 16	7.6 (1.09)	7.94 (1.17)	7.54 (1.06)	7.99 (1.21)	7.67 (0.92)	8.10 (0.99)
**Recent memory** (*M*, SD) Max score = 36	8.52 (3.66)	7.41 (3.47)	8.71 (4.12)	8.18 (3.75)	10.15 (5.0)	6.43 (2.11)
**Recent spatial** (*M*, SD) Max score = 24	2.19 (1.68)	2.65 (1.92)	2.43 (1.66)	3.11 (2.38)	2.78 (1.80)	2.62 (1.83)

Abbreviations: F, female; M, male; SPAM, Spatial Navigation and Memory Questionnaire; SD, standard deviation.

## 4 DISCUSSION

The SPAM was developed as a clinically relevant assessment of spatial navigation, memory, and attention, with separate subscales for premorbid versus recent functioning. We provide normative data from healthy older adults > 65 years of age to facilitate use in clinical practice. We evaluated the factor structure and psychometric properties of the SPAM in a sample of cognitively healthy individuals and identified a stable two‐factor structure in both subscales of the SPAM, reflecting distinct but related constructs of spatial navigation and memory. These findings, supported by good model fit indices and acceptable internal consistency, suggest that the SPAM adequately captures domains of spatial navigation and memory function.

Moderate correlations between the spatial navigation and memory factors (lifetime: *r *= .51; recent: *r = *.36) reflect conceptual overlap while supporting their distinct domains. Confirmatory factor analysis supported this two‐factor structure in both subscales. Despite significant chi‐squared estimates, which were expected given the large sample, model fit indices were acceptable. Factor loadings of SPAM items were significant and consistent with conceptual expectations. In particular, spatial navigation items in the lifetime subscale showed high loadings, suggesting stronger differentiation of this construct when retrospectively appraised.

We showed statistically significant, though weak, correlations between recent spatial navigation self‐ratings and both egocentric and allocentric error measured by the VST, indicating that poorer self‐reported recent spatial navigation ability was associated with worse objective spatial navigation performance. Difference scores between lifetime and recent subscale scores, reflecting perceived change in spatial navigation ability over time, also correlated with VST performance. As expected, no significant relationships were observed between SPAM memory items and VST performance, reinforcing the independence of the two SPAM subscales. These findings provide support for the convergent and divergent validity of the spatial navigation component of the SPAM, while also concurrently demonstrating convergent validity of the VST. Nonetheless, the weak correlations between spatial navigation scores on the SPAM and VST suggest that although both measures assess the same cognitive construct, they may be capturing different aspects of spatial navigation. Further, the VST may be more sensitive and specific to subtle performance changes before they are subjectively perceived on self‐report measures like the SPAM.^31^, ^32^ Self‐report and objective measures are often used to assess the same construct; however, there have been consistent reports of weak correlations between the two types of measurement across multiple psychological domains.^33^ This may likely be influenced by subjective judgments about self‐performance,^34^, ^35^ rather than the performance itself. For example, women show discrepancies between their subjective ratings and objective performance on measures of spatial navigation, relative to men.^34^, ^36^, ^37^ The use of a healthy sample with limited deficits on the SPAM and VST may have also contributed to the weak associations observed.

Once validated in a clinical sample, the SPAM may offer a practical and time‐efficient tool for clinicians to assess spatial navigation and memory ability. The structure of the SPAM also allows interpretation of perceived change, with premorbid assessments captured through the lifetime subscale, and current self‐perceptions in the recent subscale. While the broad temporal scope of the lifetime subscale—which asks participants to reflect on their spatial navigation and memory abilities over their lifetime—potentially introduces recall bias, this approach was intentionally taken to estimate premorbid functioning, as narrowing the timeframe to the last 5 to 10 years may inadvertently capture early cognitive decline or age‐related changes. By using the lifetime framing, the SPAM is better positioned to detect those who report longstanding difficulties with memory or spatial navigation which can have meaningful implications for clinical interpretation and decision making (e.g., individuals who have longstanding difficulties with spatial navigation). The difference scores between lifetime and recent abilities may also be clinically useful, such that larger scores indicate greater perceived cognitive decline. In such cases, re‐administration of the SPAM or referral for more detailed neuropsychological testing may be recommended to monitor and track perceived and objective changes in spatial navigation and memory function.

Importantly, the SPAM incorporates items that reflect safeguarding concerns as well as attentional memory items, which are lacking in existing questionnaires. The safeguarding items are a potentially useful subset of questions for clinical interpretation, alerting clinicians to increased risk with activities of daily living, identifying those who may benefit from further evaluation, and informing early interventions and support planning. While the safeguarding items are important for identifying clinically relevant difficulties, their relevance in a healthy older adult sample may be limited and may have influenced the associations reported in the current study. Another potentially clinically significant subset of questions included in the SPAM are attentional memory items (Q5 and Q6 in the lifetime subscale). Attentional deficits can impact memory encoding and retrieval,^38^ and are a common and early presenting complaint.^39^ Early subjective memory complaints may be driven by subtle attentional deficits, and the SPAM may help identify whether perceived memory complaints may in fact stem from attentional deficits. Higher endorsement of attentional memory items may also inform care planning and interventions.

Importantly, our study establishes normative data to interpret scores in a range of assessment contexts for different sex and age groups in healthy older adults. We observed no large variability or decline in SPAM scores in healthy older adults between age groups, suggesting that perceptions of spatial navigation and memory abilities may not change with normal healthy aging. This is consistent with evidence suggesting that healthy older adults do not typically self‐report spatial navigation difficulties.^4^ These normative scores provide a reference for future validation studies in clinical populations, which will be necessary to determine the extent of the SPAM's diagnostic utility.

The current study has multiple strengths. First, we used a large, pre‐existing data set, which afforded sufficient power to conduct both exploratory and confirmatory factor analysis of the SPAM. The SPAM uniquely captures both premorbid (lifetime) and recent functioning within the same brief self‐report questionnaire. This feature allows within‐subject comparison of perceived spatial navigation and memory changes over time, which is particularly valuable in aging contexts in which retrospective estimation of premorbid function is often clinically important but is rarely assessed systematically. While some techniques exist to estimate premorbid functioning, judgments are usually based on a single evaluation using demographic variables and/or performance measures of crystallized intelligence,^40^, ^41^ rather than spatial navigation or memory abilities.^42^ Furthermore, other commonly used measures of spatial navigation were originally developed and validated in college students.^12^ By contrast, the SPAM was developed for and evaluated in a large older adult sample. Last, the remote administration of the SPAM and VST in this study aligns with emerging digital approaches to spatial navigation assessment for early detection of AD.^43^

One key limitation of the current study is the absence of a clinical group (e.g., dementia), which limits conclusions regarding diagnostic sensitivity of the SPAM. However, with the aim of establishing normative data, it is necessary and appropriate to first evaluate a healthy non‐clinical sample, providing a baseline from which meaningful deviations in clinical samples can be identified. In addition, as participants in the current study were from the DECISION study, which focused on cognition and driving behavior,^19^, ^20^, ^21^ our sample did not include older adults in the UK who were not current drivers. Therefore, future studies with more representative healthy adult and clinical cohorts are needed to further validate the SPAM and establish cut‐off scores for clinically meaningful spatial navigation and memory impairments. The primary focus and novelty of this study was the spatial navigation subscale validity; however, future work should assess convergent validity of the memory component of the SPAM with an objective memory assessment. Previous studies have established convergent validity between subjective and objective memory performance,^44^, ^45^, ^46^ indicating that similar patterns may reasonably be expected for the memory subscale of the SPAM. Furthermore, as no measure of global cognitive function was included, we are unable to directly account for overall cognitive status or its potential contribution to task performance. Finally, due to limited range in education level, norms could not be stratified by education. Future studies should aim to recruit a wider range of participants from different educational backgrounds as it may impact spatial navigation and memory ability.^47^, ^48^, ^49^, ^50^

The SPAM demonstrates a clear and interpretable factor structure, acceptable model fit, and preliminary evidence of convergent and divergent validity with an objective measure of spatial navigation. The dual focus on lifetime (premorbid) and recent memory and spatial navigation functioning, inclusion of clinically relevant items, and normative benchmarks based on a large sample of healthy older adults support its value in both research and clinical settings. Future validation in clinical samples including those with mild cognitive impairment and dementia will further clarify its diagnostic application.

## CONFLICT OF INTEREST STATEMENT

Michael Hornberger was involved in the development of the Spatial Navigation and Memory (SPAM) questionnaire. The SPAM is not commercialized and there are no financial conflicts of interest. Remaining coauthors have no conflicts of interest to declare. Author disclosures are available in the Supporting Information.

## CONSENT STATEMENT

All human subjects provided informed consent.

## Supporting information




**Supporting Information**:dad270441‐sup‐0001‐ICMJE.pdf

**Supporting Information**: dad270441‐sup‐0002‐SuppMat.docx
