# Supplementary material for: Development and validation of the Spatial Navigation and Memory Questionnaire (SPAM)
Source: Alzheimers Dement (Amst). 2026 Aug 11;18(3):e70441. doi: 10.1002/dad2.70441 (PMC13461775; doi:10.1002/dad2.70441)
Supplement: Supplementary file 2 [file DAD2-18-e70441-s001.docx]

Supplementary Table 1. Items comprising the Spatial Navigation and Memory Questionnaire (SPAM)

|  | **Domain** | **Question** |
| --- | --- | --- |
| 1 | Lifetime memory | I always had a good memory for events |
| 2 | Lifetime memory | I always had a good memory for names |
| 3 | Lifetime memory | I always had a good memory for faces |
| 4 | Lifetime memory | I rarely needed to keep lists to remember things |
| 5 | Lifetime memory | I rarely got distracted when trying to remember something |
| 6 | Lifetime memory | My mind rarely drifted during conversations or meetings |
| 7 | Lifetime spatial | I have always been good at finding my way around, even without maps or GPS |
| 8 | Lifetime spatial | I have always been good at giving directions |
| 9 | Lifetime spatial | I would rarely get lost in new places, for example on holiday or in an unknown town |
| 10 | Lifetime spatial | I usually followed landmarks when finding my way around |
| 11 | Lifetime spatial | I had an internal map (in my head) of my environment when finding my way around |
| 12 | Lifetime spatial | I rarely got disoriented as to my location |
| 13 | Recent memory | I have difficulty learning new information |
| 14 | Recent memory | I have difficulty remembering recent events |
| 15 | Recent memory | I have difficulty remembering events from long ago |
| 16 | Recent memory | I struggle to remember appointments |
| 17 | Recent memory | I forget what I was looking for when going to another room |
| 18 | Recent memory | I struggle to remember where things belong in the house |
| 19 | Recent spatial | I have been unsure where the rooms are in my home |
| 20 | Recent spatial | I have been unsure how to get to familiar places in my neighbourhood |
| 21 | Recent spatial | I avoid going to new places as I am not sure how to find my way there |
| 22 | Recent spatial | I avoid going to places in the dark, as I am getting lost more easily |
| 23 | Recent spatial | I am looking more for landmarks, like church towers or distinctive buildings, to find my way around |
| 24 | Recent spatial | If there is a roadblock or deviation in my journey, I have problems finding another route without help |
| 25 | Recent memory* | I have forgotten to switch off an appliance (such as the oven) |
| 26 | Recent memory* | I have forgotten to eat or drink, without being reminded |
| 27 | Recent memory* | I have forgotten to take my medication, without being reminded |
| 28 | Recent spatial | I have thought that I might not find my way back to my home when out |
| 29 | Recent spatial | I have been disoriented as to my location when driving |
| 30 | Recent spatial | I have relied on my GPS to navigate |

**Safeguarding item*

**Supplementary Text A: Virtual Supermarket Test (VST)**

The VST is a passive virtual reality test of spatial navigation administered through the NeurOn online remote testing platform (https://neuropsychology.online/). On each of the 14 trials, participants were presented with a first-person perspective video of a trolley (shopping cart; see Supplementary Figure 1a) moving through a virtual supermarket Specifically, participants were instructed to imagine they were standing behind the trolley pushing it through the supermarket and that the trolley would take different turns, moving up and down different aisles of the supermarket. At the end of each trial, the trolley stopped at a supermarket location (see Supplementary Figure 1b) and participants were asked to provide egocentric and allocentric navigation responses:

1) Egocentric navigation: Using a 360-degree digital dial (see Supplementary Figure 1c), participants were prompted indicate the direction of their starting point (entrance to the supermarket) relative to where they had stopped in the video. For each egocentric trial, the error scores were calculated as the absolute degree difference between the correct and participant-reported starting direction.
2) Allocentric navigation: Following the egocentric navigation response, a map of the supermarket was displayed (see Supplementary Figure 1d) and participants were prompted to draw the path of the supermarket trolley presented during the video. Allocentric error was defined as the Euclidean distance between the endpoint of the participant's reconstructed path and the correct (true endpoint) location in the virtual supermarket.

Mean egocentric and allocentric errors were calculated and converted to z-scores for standardised comparison. Higher error scores indicate worse egocentric and allocentric navigation error. Outlier trials were identified and removed based on z-scores ≥ +3 or ≤ –2.99 across the entire sample (occurred in ~1.5% of trials).

**Supplementary Figure 1.** Representative screenshots from the VST showing the participants’ view at the a) beginning and b) end of a trial, c) the egocentric navigation response and d) the allocentric navigation response.

**Supplementary Text B: Age and sex differences in SPAM subscales**

*Lifetime subscale*

For the spatial navigation subscale there was a significant main effect of sex F(1, 934) = 12.94, *p* <.001, n^2^p =.014, where males scored lower than females indicating better spatial navigation. There was no significant main effect of age group F(2,934) = 0.30, *p* = .740, n^2^p = .001 and no significant sex*age group interaction F(2,934=0.25, p = .782, n^2^*p* = .001.

For the memory subscale there was no significant main effect of sex F(1, 934) = 0.64, *p* = .425, n^2^p = .001, age groups F(2,934) = 2.11, *p* = .122, n^2^p = .004 and no significant sex*age group interaction F(2,934) = 0.14, *p* = .873, n^2^p = .000.

*Recent subscale*

For the spatial navigation subscale there was a significant main effect of age group F(2,934) = 3.49, *p* = .031, n^2^p = .007. Post-hoc comparisons showed that the 70-79 group scored significantly higher than the 60-69 year old group (mean difference -0.35, SE = 0.13, *p* = .025). There was no significant main effect of sex F(1,934) = 2.85, *p* = .092, n^2^p = .003 and no significant sex*age interaction F(2.934) =1.31, *p* = .270, n^2^p = .003.

For the memory subscale there was a significant main effect of sex F(1, 934) = 22.87, *p* <. 001, n^2^p =.024 where males scored higher than females indicating poorer memory. There was no significant main effect of age group F(2,934) = 1.71, *p* = .182, n^2^p = .004. The sex*age group interaction was significant F(2,934) = 4.59, *p* = .0.10, n^2^p = .010 where females scored significantly lower than males across all age groups, with the largest difference observed in the 80-89 years group (mean difference 3.72, p = .003).

**Supplementary Text C: Difference between recent and lifetime subscale scores**

To explore changes in recent and lifetime spatial navigation and memory abilities, difference scores were calculated for each participant by subtracting lifetime subscale scores from recent subscale scores for spatial navigation and memory items on the SPAM. Means and standard deviations for these difference scores are reported in Supplementary Material Table 2. Larger difference scores indicate a greater perceived decline in spatial navigation or memory ability.

To examine whether difference scores in spatial navigation and memory were associated with VST performance, correlations between difference scores and egocentric and allocentric error were assessed. A weak positive correlation was shown between the difference score for spatial navigation items on the SPAM and allocentric error (*r* = 0.154, *p* =.001) and egocentric error (*r* = 0.117, *p* = .014). No significant correlations were found between the difference score for memory items on the SPAM and either allocentric or egocentric error.

| Supplementary Table 2. Difference scores for spatial navigation and memory items on the SPAM | | |
| --- | --- | --- |
|  | Spatial navigation  difference score | Memory  difference score |
| N | 445 | 445 |
| Mean | -5.13 | -2.88 |
| Standard deviation | 2.31 | 4.65 |
| Minimum | -12 | -12 |
| Maximum | 5 | 17 |

Supplementary Table 3. Sample characteristics of the EFA and CFA samples

|  | **Age (M, SD)** | **Sex (M/F)** | **Highest level of education (n, %)** |
| --- | --- | --- | --- |
| **EFA sample** (n=470) | 71.3 (5.1) | 218/252 | < secondary school n=1 (0.2%)  Secondary school: O’level/GCSE n=50 (10.6%)  Secondary school: A level n=18 (8.1%)  Vocational diploma/certificate/apprenticeship n=110 (23.4%)  Bachelor’s degree n=162 (34.5%)  Master’s degree n=77 (16.4%)  PhD n=32 (6.8%) |
| **CFA sample** (n=470) | 70.9 (4.9) | 201/269 | < secondary school n=3 (0.6%)  Secondary school: O’level/GCSE n=31 (6.6%)  Secondary school: A level n=29 (6.2%)  Vocational diploma/certificate/apprenticeship n=111 (23.6%)  Bachelor’s degree n=165 (35.1%)  Master’s degree n=97 (20.6%)  PhD n=34 (7.2%) |
| *Note*. EFA= exploratory factor analysis; CFA= confirmatory factor analysis; GCSE= general certificate of secondary education; O’level= ordinary level; A’level= advanced level. | | | |
